# Bovine Leukemia virus (BLV) and risk of breast cancer: a systematic review and meta-analysis of case-control studies

**DOI:** 10.1186/s13027-020-00314-7

**Published:** 2020-07-22

**Authors:** Alireza Khatami, Ali Pormohammad, Rana Farzi, Hassan Saadati, Maryam Mehrabi, Seyed Jalal Kiani, Saied Ghorbani

**Affiliations:** 1 Department of Virology, Faculty of Medicine, Iran University of Medical Science, Tehran, Iran; 2 Department of Biological Sciences, University of Calgary, Calgary, AB Canada; 3 Department of Virology, Faculty of Medicine, Shiraz University of Medical Science, Shiraz, Iran; 4 Department of Epidemiology, School of Public Health, Iran University of Medical Sciences, Tehran, Iran; 5 Department of Microbiology, Karaj Branch, Islamic Azad University, Karaj, Iran; 6 Student Research Committee, Iran University of Medical Sciences, Tehran, Iran

**Keywords:** Breast cancer, Bovine leukemia virus, BLV, Meta-analysis

## Abstract

**Background:**

Breast cancer is reported as one of the most common cancers among females worldwide. Infectious agents especially viruses have been considered as role players in the development of breast cancer. Although some investigations suggest an association between bovine leukemia virus (BLV) and breast cancer, the involvement of this virus as a risk factor remains controversial. The present study aimed to find out any possible association between BLV and breast cancer through conducting a systematic review and meta-analysis.

**Methods:**

Systematic literature search was performed by finding related case-control articles from the PubMed, Google Scholar, Web of Science, Scopus, and EMBASE databases. The heterogeneity and the multivariable-adjusted OR and corresponding 95% CI were applied by meta-analysis and forest plot across studies. All statistical analyses were performed using Stata 14.1.

**Result:**

Based on a comprehensive literature search, 9 case-control studies were included for meta-analysis. The combination of all included studies showed that BLV infection is associated with an increased risk of breast cancer [summary OR (95% CI) 2.57 (1.45, 4.56)].

**Conclusion:**

This is the first meta-analysis to analyze a potential association between BLV infection and the risk of breast cancer. Control of the infection in cattle herds and screening of the milk and dairy products may help to reduce the transmission of the virus to humans.

## Introduction

Breast cancer is known as the second common cancer (after skin cancers) in females in the United States [1, 2]. According to the World Health Organization estimates, about 2.1 million women are diagnosed with breast cancer annually [3]. According to the data from cancer statistics, 2019, approximately 268,600 cases are diagnosed each year in the United States. This is about 30% of all new cases of diagnosed cancers in women. Moreover, breast cancer is considered as the second deadliest cancer (after lung cancer) in female population with annual 627,000 deaths in the world [3] and 41,760 deaths in the United States. This represents 15% of all cancer deaths in women [1]. Total incidence rate of cancer in women has been constant over the last decades, but an increase in the incidence of breast cancer has been reported from 2006 to 2015 [1]. Due to the high incidence and mortality rates of breast cancer, it is important to find new risk factors that might be associated with the stimulation and development of breast cancer. Evidently, breast cancer is a multifactorial disease and a large number of risk factors could play important roles in the development of the disease [3]. Currently, the main cause of breast cancer is not known, however, genetics, malnutrition, lifestyle, aging, tobacco and alcohol use, obesity, and infectious agents are considered as risk factors [4]. Based on the International Agency for Research on Cancer, infectious agents, such as viruses are associated with the development and progression of 15–20% of tumors, in general [5].

Among viruses, Human papillomavirus [6] and Mouse Mammary Tumor Virus-Like Virus (MMTV-LV) [7] infections have been associated with the development of human breast cancer. Another study showed that Epstein-Barr virus (EBV) infection may have a potential role in the breast cancer development, as well [3]. Moreover, several recent studies have suggested a possible relationship between BLV (Bovine leukemia virus) and breast cancer [8, 9].

BLV belongs to the *Retroviridae* family. The virus is further classified in Orthoretrovirinae subfamily, and deltaretrovirus genus which is considered as a possible zoonotic infection [9]. The main hosts for BLV is cattle but it can infect other animal species such as water buffalo, sheep, alpaca, rabbits, rats, pigs, and goats, as well. The prevalence of BLV infection is high in cattle herds and varies from about 39 to 100% in beef and dairy herds, respectively. Although BLV can easily transmit through infected blood and milk, it causes disease in less than 5% of infected cattle [8, 9]. BLV is associated with chronic lymphatic leukemia and the infection of mammary cells might be associated with breast tumor in the hosts. The BLV tax protein has some regulatory functions (transcription activator) and could be associated with transformation through inhibition of DNA repair system and disruption of tumor suppressor genes [10]. The mechanism of BLV transmission to humans is not known, however, raw milk consumption can transmit the virus from cattle to human population [11]. The study by Buehring et al. showed that the genome and antibodies against the capsid protein (P24) of BLV can be found in female blood samples [8, 9]. Buehring et al. detected the BLV DNA in the breast tissue of 80% of women with breast cancer in compare with 41% of negative control group [12].

Because there are some discrepancies regarding the association between BLV and breast cancer, the association of this virus as a risk factor for breast cancer development remains controversial. The present study aimed to find out any possible association between BLV and breast cancer through conducting a systematic review and meta-analysis.

## Method

### Search strategy

This study was done based on the PRISMA (Preferred Reporting Items for Systematic Reviews and Meta-Analyses) guidelines. Systematic literature search was conducted by independent reviewers and all related articles from global databases were collected from January 1995 to January 2020 [13]. All case-control studies that investigated the BLV infection in breast cancers were collected from well-known databases such as the PubMed, Google Scholar, Web of Science, Scopus, and EMBASE. The Mesh-indexed keywords, including “breast cancer”, “Bovine Leukemia virus” OR “BLV” and their synonyms were used. Additional related articles were assessed by reviewing the references of the selected publications and reviews as well as the excluded ones.

### Study selection

All the articles were imported into Endnotes software version X7and the duplicates were removed. Then, the title and abstract of the articles were analyzed to exclude all irrelevant publications. Full-texts of the remaining articles were reviewed and disagreements between the reviewers were resolved. All remaining articles were included. All those responsible for searching and filtering the articles were contacted by email and other Virtual Contact Methods.

### Eligibility criteria

The following criteria were applied for the selection of qualified studies in this research:

All BLV case-control and prevalence studies published in English language, publication date between 1995 to January 2020, availability of the full-text, and application of standard assays for the detection of BLV nucleic acid and antigens, including RT-PCR, Immunohistochemistry (IHC), In situ hybridization (ISH), ELISA (enzyme-linked immunosorbent assay) Nested PCR and In situ PCR assays.

The exclusion criteria were as follows:

Studies published in languages other than English, studies regarding breast cancer association with other viruses rather than BLV, studies examining the prevalence of BLV infection in male patients, article types of systematic review, meta-analysis, case report, letter to the editor, and conference abstracts.

### Data extraction

Data extraction for the selected studies carried out by two independent reviewers. The extracted data included: the author’s name, year of publication, country, geographical area, type of study, sample type, target gene, the sample size, number of BLV positive samples, mean age, and detection method.

### Quality assessment

After selection of the relevant studies in terms of the title and contents, the Newcastle- Ottawa assessment scale (NOS) was used to evaluate the quality of the articles. The NOS is used to evaluate three quality parameters of selection, Comparability, and Exposure. Each step has some questions and the score is assessed based on the given stars (*). These questions are related to the case definition, case and control selection, definition of control, comparability of case and control, detection method and the type of control (1 star for each question). In prepared checklist, the highest score was 10 and the lowest acceptable score was 6. Finally, the articles with a score of at least 6 were selected and data was extracted (Table 1).

**Table 1:** Characteristics of studies included in the current study

study	Publication year	Sample collection date	country	continent	Study-design	Sample type	Detection target	Case	Case positive	Control	Control positive	Detection method	Age control	Age case	NOS score	ref
Giovanna	2013	Since 2006	Colombia	South America	Case–control	FFPE	Gag	53	19	53	24	PCR	52.2	46.4	8	[14]
Schwingel	2019	2015–2017	Brazil	South America	Case–control	FFPE	Tax	72	22	72	10	Nested PCR	38	52.5	9	[15]
Buehring	2007	NR	US	North America	Case–control	FFPE	Tax	110	65	103	30	In situ PCR	NR	NR	9	[16]
Buehring	2015	2002–2008	US	North America	Case–control	FFPE	Tax	114	67	104	30	In situ PCR	48.7	55.9	9	[17]
Baltzell	2017	2009–2011	US	North America	Case–control	FFPE	Tax	61	35	103	20	In situ PCR	54.4	54.59	9	[18]
Buehring	2017	1995–2010	Australia	ocenia	Case–control	FFPE	Tax	50	40	46	19	In situ PCR	49.59	55.08	9	[12]
Lawson and Glenn	2017	NR	Australia	ocenia	Case–control	FFPE	Tax	22	20	17	6	In situ PCR	36.4	56.1	8	[19]
Khalilian	2019	2017–2018	Iran	Middle East	Case–control	FFPE and Blood	Tax	172	52	200	60	In situ PCR	47.5	53	9	[11]
Khalilian	2019	2017–2018	Iran	Middle east	Case–control	FFPE and Blood	Gag	172	14	200	16	Nested PCR	47.5	53	8	[11]

*FFPE* Formalin-fixed paraffin-embedded, *PCR* Polymerase chain reaction, *NOS* Newcastle- Ottawa assessment scale

### Statistical analysis

The heterogeneity of study populations was assessed by random-effects meta-analysis framework and for visual inspection of the multivariable-adjusted OR and corresponding 95% CI a forest plot was produced across studies. The logarithm of the odds ratio and its associated standard error was used in this meta-analysis. Der-Simonian and Laird method was also used to estimate pooled OR with its corresponding 95% CI [20]. The Cochran’s Q statistic (*P* < 0.10) was applied to check the heterogeneity between studies [21] and quantified using the I^2^ statistic [22]. The I^2^ > 50% was considered as high heterogeneity. Sources of heterogeneity were identified using meta-regression and subgroup analyses. A combination of the visual inspection of funnel plots [23], Begg’s test and Egger’s test [24] were performed to investigate the presence and the effect of publication bias. The extent of inferences dependency on a particular study or group of studies was detected using the sensitivity analysis. Two-tailed statistics and the significance level of less than 0.05 were considered for all analyses, except the heterogeneity test with significance level of less than 0.1. All statistical analyses were performed using Stata 14.1 (Stata Corp, College Station, TX, USA).

## Result

### Study selection

**Fig. 1:**
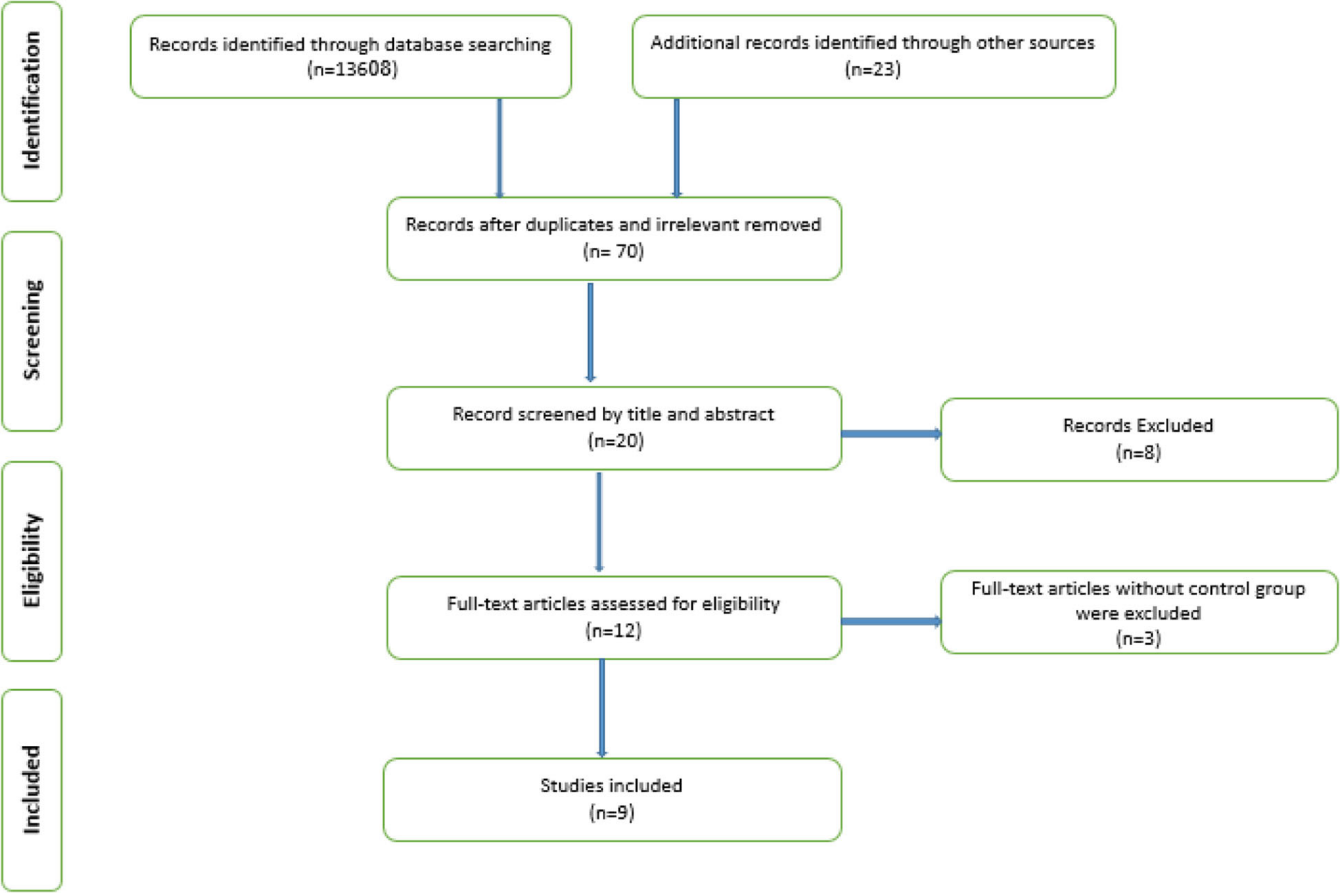
PRISMA flowchart of included studies

The PRISMA flow chart was used to illustrate the results of the literature search and selection process (Fig. 1). Altogether, 13,631 potentially relevant articles were initially selected. Duplicates and irrelevant studies were removed. Of 70 remained articles, 61 studies were excluded for following reasons: did not present sufficient data, studies without control group, and studies that were not in English. Finally, 9 articles were included in the meta-analysis.

### Study characteristics

The characteristics of the selected studies of this analysis are summarized in Table 1. Nine case-control studies were selected for further analysis of which 3 were from the North (US) and Caribbean America, 2 studies from Western pacific region (Australia), 2 studies from Eastern Mediterranean region (Iran), and 2 studies from south America (1 study from Brazil and the other from Colombia Formalin-fixed paraffin-embedded (FFPE) tissue samples were investigated in all the studies and blood samples were analyzed in 2 studies as well. Methodologically, in-situ PCR, nested-PCR, and conventional PCR tests were performed in 8, 3, and 1 studies, respectively. Virus detection was performed based on the detection of viral gag gene in 2 studies and viral tax gene was investigated in the rest.

### Characteristics of participant

In total, there were 826 cases and 898 individuals as control group. The median age in case and control groups was ranged between 46.4–56.1 and 36.4–54.4, respectively. The sample size in case and control groups was ranged between 22 and 172 and 17–200, respectively. Publication date of the articles ranged from 1995 to 2020.

### Risk of breast cancer in association with BLV

**Fig. 2:**
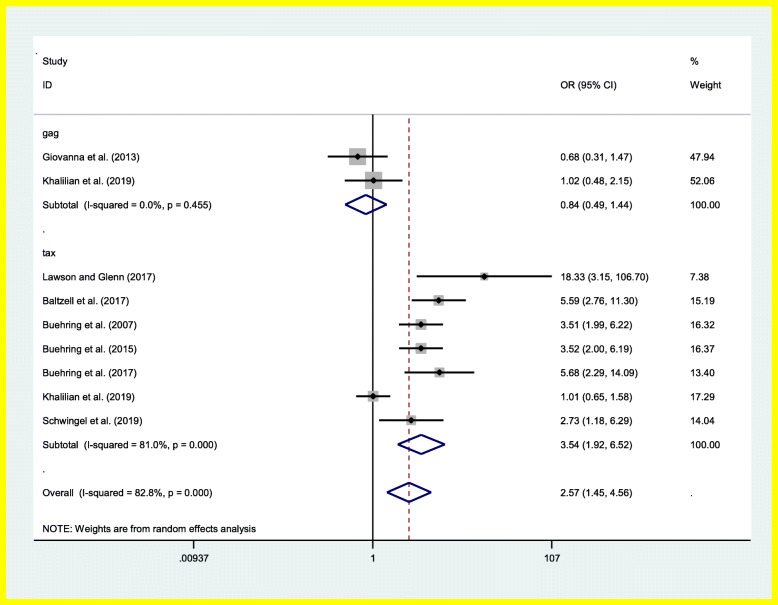
Association between BLV and breast cancer risk in case-control studies based on detection-target

Based on Cochran’s Q and I^2^ statistics, significant heterogeneity was found between the selected studies (Q = 46.62, df = 8, *P* < 0.001, I2 = 84.8%) and supported the use of a random effects model. To study heterogeneity sources, meta-regressions were carried out based on the sample type, the detection target, the detection method and the sample size. The results are presented in Table 2. In addition, a subgroup meta-analysis was performed by detection-target. The results of each study and the total summary results for the nine selected case-control studies of the BLV infection and breast cancer, according to detection-target are shown in Fig. 2.

**Table 2:** The result of meta-regressions

Variables	Coefficient	Standard error	T	p value	Lower 95%	Upper 95%
Sample type	0.07	1.38	0.05	0.96	−3.78	3.92
Detection target	−1.35	0.68	−1.98	0.11	−3.24	0.54
Detection method	0.23	0.66	0.35	0.74	−1.61	2.07
Sample size	0.004	0.005	−0.79	0.47	−0.02	0.01

### Overall analysis of the used target gene for BLV detection

Analysis of the 9 case-control studies revealed that BLV infection was associated with an increased risk of breast cancer [summary OR (95% CI) 2.57 (1.45, 4.56)]. Of the 9 studies, detection of BLV infection was performed via identification of the tax gene in 2 studies, while the gag gene was the target in the other 7 studies (Table 3). Based on the gag gene, there were 225 cases and 253 individuals as the control group. Based on the tax gene, there were 601 cases and 645 individuals as the control group. A sensitivity analysis was performed by successive removal of individual studies at a time to evaluate the effect of each study on pooled results. A significant positive association [range of summary ORs 2.26–3.02] was consistently found between BLV virus and breast cancer which did not alter the pooled results. This indicates that the meta-analysis performed in this study was powerful.

**Table 3:** Association between breast cancer and BLV detection-target gene

Gene target	No of studies	Odds ratio (95%Cl)	Heterogeneity chi squared	Odds ratio two-tailed p value	Heterogeneity test, *p* value
Gag	2	0.84 (0.49–1.44)	0.56	0.51	0.45
Tax	7	3.54 (1.92–6.52)	31.64	< 0.001	< 0.001
total	9	2.57 (1.92–6.52)	46.62	0.001	< 0.001

## Discussion

The main etiology of breast cancer is unknown and several factors are associated with cancer development and progression. Previous studies have reported that infectious agents especially viruses could be related to breast cancer development [3]. In some meta-analysis studies of the association of papillomavirus, EBV, and MMV with breast cancer, the results showed that epidemiologically, the prevalence of these viral infections in patients with breast cancer was higher in comparison with the control group [3, 6, 25]. Several cross-sectional and case-control studies indicated that BLV virus infection may be associated with breast cancer, as well [12, 18].

To the best of our knowledge, this study is the first meta-analysis investigating the association between BLV and breast cancer. In this study, 9 case-control articles investigating the association between BLV infection in patients with breast cancer and the control group were examined on blood samples and FFPE tissue samples. Present study showed that the prevalence of BLV infection, based on the studies that used In-situ PCR, Nested PCR and conventional PCR tests, was higher in patients with breast cancer in comparison with controls.

Overall, the 9 case-control studies showed that BLV infection was associated with an increased risk of breast cancer [summary OR (95% CI) 2.57 (1.45, 4.56)] and this indicates that the infection with BLV may be a risk factor for breast cancer. Although no association was reported in two studies that used BLV gag gene as the target for PCR amplification, this might be due to either small sample size or high frequency of mutations in this gene. In fact, deletion of the Gag, Pol, and Env segments of the genome has been reported in advanced stages of leukemia’s and lymphomas to more likely provide the virus with the ability to escape host’s immune responses. This may lead to lower detection levels of BLV infection in assays that target these regions of the genome [9, 26].

**Fig. 3:**
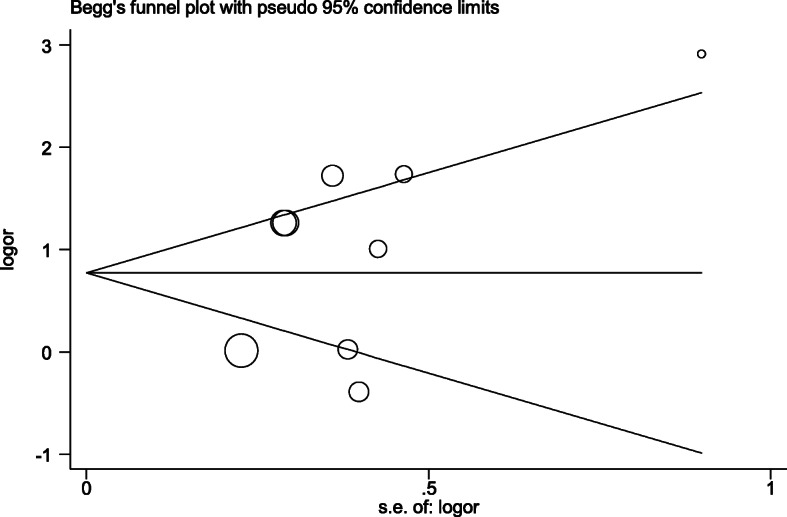
Funnel plot for estimation of publication bias

In order to evaluate the publication bias, assuming that small studies may be more susceptible to publication bias in comparison to larger studies, a visual inspection of the funnel plot and statistical tests were applied. The results presented in Fig. 3 revealed no publication bias for the association between the BLV and risk of breast cancer (data not shown; *P* = 0.297, for Begg’s adjusted rank correlation test and *P* = 0.237 for Egger’s regression asymmetry test). Moreover, the trim-and-fill method was unable to find any missing study.

Recent studies have shown that BLV receptors exist on different cell types and the virus can enter different tissues. The tax gene in BLV is responsible for oncogenic activity and inhibits the mechanism of cell excision repair system and leads to oxidative damage in the cell. This may be associated with various cancer types such as lung and breast cancers. Olaya-Galan et al. showed that BLV-contaminated milk can transmit the virus horizontally to other hosts [27]. On the other hand, milk and dairy products are one of the most important sources of human nutrition, and the raw consumption of these products can be involved in virus transmission to humans [11].

Language barriers, limitation to female-based studies, and excluding the studies without a control group were the important limitations of this study.

## Conclusion

This is the first meta-analytic study to find the association between BLV infection and the risk of breast cancer. Control of the infection in cattle herds and screening of the milk and dairy products may help to reduce the transmission of the virus to humans. However, more research are needed to have a better understanding regarding the involvement of this virus in the etiology of breast cancer.

## Data Availability

All data used and analyzed are available from the corresponding author.
